# Advancing educational equity in rural China: the impact of AI devices on teaching quality and learning outcomes for sustainable development

**DOI:** 10.3389/fpsyg.2025.1588047

**Published:** 2025-11-05

**Authors:** Ronghui Chen, Yuanyuan Wu, Zhe Chen, Peng Zhou

**Affiliations:** ^1^School of Journalism and Communication, Hangzhou City University, Hangzhou, China; ^2^School of Graduate Studies, Lingnan University, Hongkong, China; ^3^FutureFront Interdisciplinary Research Institute, Huazhong University of Science and Technology, Wuhan, China

**Keywords:** sustainable, AI assisted teaching, educational equity, education investment, sustainable educational growth

## Abstract

This study investigates the impact of AI-assisted teaching on teaching quality and learning outcomes in rural schools in China, aiming to promote educational equity and sustainable educational growth. Through questionnaire surveys of 268 teachers and controlled experiments in 12 schools (4 urban, 8 rural), we assess whether AI-integrated Mixed Reality (MR) devices can enhance educational experiences in resource-constrained environments, supporting sustainable development. The research integrates the Technology Readiness Index (TRI), Innovation Diffusion Theory (IDT), and Technology Acceptance Model (TAM) to propose a comprehensive framework for studying teachers’ acceptance of these devices. Results indicate that AI-assisted teaching significantly improves teaching quality and learning outcomes, particularly in natural science courses, with rural schools showing greater gains (15.69% score improvement vs. 10.27% urban). Education investment in such technologies can reduce urban–rural disparities. Future research should explore subject-specific applications, strengthen teacher training, and enhance technical support to achieve educational equity and sustainable educational growth.

## 1 Introduction

Sustainable development in rural education faces numerous challenges, including insufficient educational resources, weak teaching staff, outdated infrastructure, and a shortage of educational funding; low education quality with unreasonable curriculum settings, outdated teaching methods, and an imperfect evaluation system; unequal educational opportunities with a significant urban–rural gap, gender discrimination, and difficulties in education for disadvantaged groups; disconnection between education and socio-economic development, insufficient vocational education and training, and employment difficulties for graduates; cultural and social factors affecting education, traditional concepts restricting progress, and low community participation; and weak awareness of environment and sustainable development, lack of environmental protection knowledge, and low efficiency in resource utilization (Arnold et al., 2005; Van Crowder et al., 1998; Zikargae et al., 2022). To address these issues, increased investment, improved teacher quality, optimized curriculum settings, promotion of educational equity, strengthened vocational education, increased social participation, and the popularization of sustainable development concepts are needed (Xue et al., 2021).

Under the framework of sustainable development, introducing AI (Artificial Intelligence) devices into rural education is of great significance (Mangione et al., 2024). It can not only significantly improve the quality of education by optimizing learning experiences through personalized learning and intelligent tutoring but also alleviate the problem of insufficient resources, achieve remote education and virtual experiments, promote educational equity, reduce the urban–rural gap, and provide support for special groups. The application of AI has driven educational innovation, cultivated students’ technological literacy and interdisciplinary capabilities, contributed to the achievement of sustainable development goals, and both supported economic growth and enhanced environmental protection awareness (Lu and Sun, 2024).

The benefits of AI in education include enhancing the personalization of teaching, optimizing the allocation of resources, enriching the learning experience, and improving the quality and efficiency of education through efficient data processing capabilities. AI enables educators to provide tailored learning paths based on the individual differences of students, allowing each student to learn at their own pace and in a manner suitable for them (Srithar and Selvaraj, 2015; Admane et al., 2024). Furthermore, AI’s data analysis capabilities can help schools better understand student performance and needs, optimize the allocation of educational resources, and provide the most effective teaching support under limited resource constraints (Frehywot et al., 2013; Rızvı, 2023). AI can also make the learning process more vivid and interactive through technologies such as simulation and virtual reality, thereby increasing students’ interest and engagement in learning (Rafiq et al., 2022).

The application of AI in education is gradually transforming teaching methods and learning approaches, especially in resource-constrained rural areas where AI has significant potential. AI, through personalized learning, can provide customized content based on each student’s abilities and progress (Iqbal, 2023), helping rural students access high-quality educational resources that are typically only available in urban schools (Tripathi et al., 2025). Furthermore, AI supports the professional development of teachers and teaching management, improving the efficiency of educational resource use, and optimizing the allocation of resources by educational decision-makers (Malczewski and Jackson, 2000; Ismaila et al., 2024). AI not only improves the quality and efficiency of teaching but, more importantly, its potential in achieving educational equity is particularly crucial, especially in narrowing the urban–rural education gap.

Urbanization, as an important component of the global modernization process, has brought about many socio-economic changes, but it has also led to the development of social inequality. Worldwide, urbanization has accelerated economic development and technological progress, but this development is often concentrated in already developed cities and regions, while remote areas and rural areas lag behind (Terluin, 2003; Carson et al., 2022). This unbalanced development model is particularly evident in China, where, since the reform and opening up, urbanization has greatly promoted the rapid growth of the national economy, but it has also exacerbated the economic and social disparities between urban and rural areas, as well as among different cities (Salvati, 2016; Jiang et al., 2022).

The field of education is also affected by the imbalance of urbanization, with the unequal distribution of educational resources exacerbating educational inequality between urban and rural areas, and among regions. Urban schools usually have more funding, more advanced teaching facilities, and a greater variety of educational resources, while rural schools often face weak teaching staff and outdated infrastructure. For example, in China, urban students usually have access to higher-quality educational resources and information technology, while rural students may lack basic educational facilities and opportunities (Du Plessis, 2014; Song, 2023).

Although urbanization has brought about issues of educational inequality, the application of AI may provide new solutions for narrowing these gaps. AI technology, through intelligent teaching platforms and resources, can offer personalized learning and virtual classrooms for students in remote areas, thus compensating for geographical and physical resource limitations. Research indicates that AI-assisted distance education projects can effectively improve the learning outcomes and engagement of students in remote areas (Adewale et al., 2024). Additionally, the application of AI in education also includes the use of data analysis to optimize the allocation of educational resources, which helps to improve the quality and equity of education.

When discussing issues of educational equity and resource allocation, different viewpoints cover various aspects from infrastructure construction to the optimization of teaching resources. Firstly, UNICEF advocates that in situations of tight funding, priority should be given to ensuring the enrollment opportunities for girls to close the gender gap in educational opportunities (Jo et al., 2019; Guglielmi et al., 2021). On the other hand, Some researchers believe that ensuring the safety of school buildings or constructing esthetically pleasing ones to attract students should take precedence, arguing that this provides a safer and more motivating learning environment (Tas, 2016; Cayubit, 2022). Furthermore, some emphasize the need to strengthen measures to combat school violence and gang activities to ensure student safety, thereby improving learning outcomes (Osher et al., 2006; Turanovic et al., 2022). There is also the view that improving teachers’ salaries and conditions is key, as this can attract more excellent teachers to join, directly enhancing the quality of teaching (Xuehui, 2018; García and Han, 2022).

However, among these diverse needs, AI stands out as a unique tool with multiple features that can effectively support various aspects of education. AI can compensate for geographical and resource inequalities through personalized learning platforms, online resources, and data-driven teaching methods. In rural areas, AI technology can help teachers provide educational content and experiences that rival those of urban students, thus helping to narrow the urban–rural education gap. Research indicates that teaching methods supported by AI tools can significantly improve the learning efficiency and academic performance of students in rural areas (Cruz-Jesus et al., 2020; Tripathi et al., 2025). Additionally, AI tools such as teacher assistant software can reduce the administrative burden on teachers, allowing them more time and energy to focus on teaching and the individual needs of students.

Especially in areas with rapid urbanization, the proliferation of AI in education provides new opportunities for achieving educational equity. Urban areas typically have more advanced technology, and the integration and application of AI are more widespread, enabling students in these areas to enjoy the educational benefits brought by AI, such as a broader range of online resources and interactive learning tools (Adewale et al., 2024). However, to ensure educational equity, it is necessary to further promote these technologies to rural and remote areas, ensuring that students in all regions can benefit from the educational innovations and enhancements brought by AI.

The pursuit of educational equity is a cornerstone of the global agenda for sustainable development, forming the very foundation upon which a just and prosperous future is built. This principle is explicitly embedded within the United Nations’ Sustainable Development Goal 4 (SDG 4), which aims to “ensure inclusive and equitable quality education and promote lifelong learning opportunities for all” (UN General Assembly, 2022). In this context, educational equity transcends mere equality—which provides the same for all—by instead focusing on fairness. It is the practice of distributing resources, opportunities, and support based on individual student needs to overcome systemic barriers, ensuring that personal or social circumstances like race, gender, or family income do not predetermine educational outcomes (Pfeffer, 2015; Carrington et al., 2022). Therefore, achieving SDG 4 is contingent upon establishing educational equity, making it a critical prerequisite for sustainable social and economic progress.

This study aims to leverage the advantages of integrating TRI, IDT, and TAM—gaining holistic insights into adoption while mitigating individual losses like TAM’s simplicity—specifically to enhance AI adoption in rural Chinese education for sustainable equity and development. Considering that AI technology has been widely introduced in urban schools, this study will assess whether AI devices can effectively improve teaching quality and learning outcomes in rural schools, thereby supporting or refuting the necessity of promoting AI devices in rural schools from the perspective of educational equity. The goal is to promote educational equity and enhance students’ future competitiveness and awareness of sustainable development through technological innovation.

Through this research, it is hoped to gain an in-depth understanding of the role and limitations of AI technology in improving the quality of rural education and achieving educational equity within the context of sustainable development, providing theoretical and practical basis for the implementation and optimization of future educational technologies. To balance resource allocation, promote educational equity, cultivate students with future skills, and contribute to the overall progress of society.

Recent research after 2021 has emphasized the role of artificial intelligence in rural education in China. The Alibaba Foundation is narrowing the digital divide through cloud-based classrooms (Alibaba-Foundation, 2025), and Xue et al. have proposed a policy framework for sustainable revitalization (Xue et al., 2021). These studies often emphasize general implementation challenges, teacher training, or resource sharing without rigorous empirical comparisons of learning outcomes. For instance, Lu and Sun explore AI-driven online training for rural teachers (Lu and Sun, 2024), and Zhao believes that the literacy of primary and secondary school teachers should be improved in order to better utilize AI in teaching (Zhao et al., 2022). Tang et al. discuss AI’s potential for equity in underserved areas (Tang et al., 2024). However, few integrate multiple acceptance theories with controlled experiments. This study advances the field by proposing a composite model to assess teachers’ sustained adoption of AI devices, while providing quantitative evidence from 16 schools (4 urban, 12 rural) on improved outcomes in mathematics and history courses. By demonstrating greater efficacy in resource-scarce rural environments, our work offers actionable insights for targeted Education sector and AI investments to foster sustainable educational equity.

## 2 Related works

### 2.1 Application of new technologies in education

Promoting educational equity through technology is one of the important directions of modern educational reform (Tang et al., 2024). With the rapid development of information technology, the use of technological means can effectively address the issues of uneven resources and unequal opportunities that exist in traditional educational models. Through online courses and remote teaching, geographical limitations can be overcome, allowing students in remote and rural areas to also receive high-quality educational resources. For example, by utilizing MOOCs (Massive Open Online Courses) (Mahajan et al., 2019; Ma and Lee, 2023) and other online learning platforms, students can access top-tier educational resources from around the world. The application of technology can also enhance the capabilities of teachers in remote areas, enabling them to provide education of equivalent quality to that in developed regions. By engaging with and learning new technological tools, teachers in remote areas can adopt more modern teaching methods. Providing real-time learning feedback and assessment tools helps teachers better understand students’ learning progress and teaching effectiveness. For example, student classroom behavior analysis systems provide feedback on students’ learning states, allowing teachers to adjust their teaching strategies in a timely manner. Students in remote areas often cannot obtain educational opportunities equal to those of urban students due to resource scarcity. Enhancing the teaching capabilities of teachers can partially compensate for this lack of resources, providing higher quality education, and thus helping to narrow the educational gap between urban and rural areas (Hanushek, 2011; Wang et al., 2024). Education is one of the important means to achieve social justice. By improving the teaching abilities of teachers in remote areas, we can ensure that all students, regardless of where they are born, have equal opportunities to learn and develop. This equal opportunity is key to enhancing the overall justice and cohesion of society.

With the development of technology, many new technologies and devices have been applied to the field of education. Smart education is an educational form that utilizes modern technologies such as artificial intelligence, data analytics, and online learning platforms to enhance teaching effectiveness and optimize learning experiences. In a smart education environment, teachers still play a crucial role (Birky et al., 2006; Gentile et al., 2023). Especially in the case of using technology to optimize the teaching process, teacher-centered applications of smart education are of significant importance to enhance teaching effectiveness.

Teachers can enhance teaching quality through various ways. Firstly, they need to understand students’ needs in-depth (Felder and Brent, 2005; van Geel et al., 2023). By understanding students’ backgrounds, interests, learning styles, and levels of ability, teachers can effectively adjust teaching strategies and content to meet students’ learning needs. Secondly, seeking feedback and evaluation is vital (Leckey and Neill, 2001; Adarkwah, 2021). Encouraging students to provide feedback and assessments helps teachers gather multiple perspectives through teaching observations, classroom records, and peer evaluations, enabling continuous improvement and enhancement of their teaching effectiveness. Thirdly, teachers need to engage in reflection and improvement (Pinsky et al., 1998; Vadivel et al., 2021). Regularly reflecting on their teaching practices, evaluating teaching effectiveness, identifying areas for improvement, and developing improvement plans are essential.

With the development of technology, teachers need to fully leverage technological support for teaching (Eden et al., 2024). Education has undergone a wave of digital transformation, and schools are integrating various technologies for innovative teaching to assist students in achieving better learning outcomes. Teachers or school administrators can closely track the learning progress of specific students, help them adjust learning goals, and provide personalized education (Bhutoria, 2022). In recent years, the development of intelligent classrooms has begun integrating artificial intelligence technologies to automatically recognize and record most learning events and behaviors, providing real-time feedback to teachers and supporting students’ adaptive learning.

The application of AI in teaching can promote educational equity, ensure that every student receives attention from teachers, improve overall teaching effectiveness, reduce educational disparities between different individuals, and promote educational equity (Viberg et al., 2024). Equal educational opportunities allow more people to access quality education and enhance sustainable development capabilities. In China, rural and remote areas are lacking in educational resources, and schools are willing to explore educational innovation and new teaching methods and technologies (Huang, 2021), which is beneficial for the continuous improvement of school education and adaptation to future development needs. Schools focus on using new technologies to improve teaching quality, which can not only provide better education for students but also benefit the school’s own development and sustainable development of society (Wang and Shih, 2022). Teachers adopting innovative teaching technologies can improve teaching efficiency and effectiveness. Educational innovation can drive the continuous improvement of the education system and better meet the talent needs for future sustainable development.

### 2.2 Theoretical frameworks in technology adoption

The intention to use new technologies in the field of education has been extensively studied. The Technology Acceptance Model (TAM) effectively examines how perceived usefulness and perceived ease of use influence the acceptance of new technologies. Many scholars have used the TAM model to study the acceptance of intelligent devices and technologies in education (Otto et al., 2024). They found that teachers’ attitudes, computer skills, and school environment are important factors in the acceptance of online learning. Some studies (Fauzi et al., 2021; Rui et al., 2023) suggest that perceived ease of use (PEoU) and perceived usefulness (PU) are important factors in the acceptance of intelligent instructional technologies. Widanengsih (2022) found that PU and PEoU are important for attitudes toward use; PEoU does not significantly influence behavioral intention; attitudes toward use significantly influence behavioral intention.

Some scholars have adopted and extended the TAM model by including more dimensions in their analysis. Teng et al. (2022) used the UTAUT model (Unified Theory of Acceptance and Use of Technology) to study factors influencing learners’ adoption of educational metaverse platforms. The study found that performance expectancy, effort expectancy, social influence, and facilitating conditions had significantly positive effects on learners’ satisfaction. Some scholars have combined the IDT (Innovation Diffusion Theory) and TAM models to study the technology acceptance of online learning (Al-Rahmi et al., 2021; Lee et al., 2011). They found that social influence and expectation confirmation affect PEoU, PU, and satisfaction. Huang’s research also revealed that the impact of social influence and external factors on users’ continued use is more significant than internal factors such as satisfaction (Huang, 2020).

The review of existing studies indicates that researchers have employed a variety of models, each with its own focus. This study attempts to assess the effective factors by integrating multiple theories. The advantage of the TAM model lies in its simplicity and strong explanatory power, which allows it to be widely applied to various technology acceptance contexts and helps in designing technology products that better meet user needs. Intelligent portable devices are not disposable products. Especially in the field of education, long-term use is a necessary condition for achieving results. The intention to continue use in the TAM model is an important factor that needs to be evaluated. We propose a composite model, in which TAM is the core. IDT emphasizes the role of innovation characteristics, communication channels, time, and social systems in the process of innovation diffusion. It provides a framework for identifying and understanding the behavioral patterns and decision-making processes of different user groups when adopting innovations. The strength of TRI lies in its ability to offer a comprehensive understanding of technology adoption behavior. The “perceived insecurity” variable in the TRI model is an important factor in the application of intelligent technology. Facial recognition (Rafika et al., 2022) and facial emotion recognition (Savchenko et al., 2022) have been applied in teaching and management. These applications require the collection of students’ image information, and due to the fact that many students are minors, there may be security risks of information leakage. This study aims to focus on the advantages of multiple models and conduct a comprehensive study on the variables related to the continuous application of intelligent technology in the field of education by integrating the IDT, TRI, and TAM models.

## 3 Methods

### 3.1 Proposed model: a combination of TRI, IDT, and TAM

The Technology Readiness Index (TRI) model is designed to test individuals’ general beliefs about technology and measure their inclination to use and accept new technologies in order to achieve goals in their homes or workplaces (Parasuraman, 2000; Kaushik and Agrawal, 2021a). Parasuraman proposed that there is a correlation between personal technology readiness and the inclination to use technology. It can be seen as a holistic psychological state, influenced by both the driving and inhibiting forces, that determines an individual’s propensity to adopt new technologies. In essence, TRI is used to measure people’s general beliefs about technology. TRI aims to measure the following variables: optimism, innovativeness, discomfort, and insecurity. Optimism reflects an individual’s positive view that certain technology will empower them with more power, versatility, and productivity. Innovativeness is set as the motivation to try new technologies, while discomfort refers to the feeling of losing control over technology and being overwhelmed by it. Alhasan proposed that insecurity primarily focuses on distrust of the technology and suspicion of its performance quality (Alhasan et al., 2023). Insecurity primarily focuses on distrust of the technology, suspicions about its performance quality, and broader concerns like data leakage beyond facial images, including student performance data (Rojas-Mendez et al., 2017; Kaushik and Agrawal, 2021).

The Innovation Diffusion Theory (IDT) (Rogers, 2003; Ho, 2022) is one of the most commonly used models in social science. It describes the factors that influence an individual’s adoption of new technologies or ideas. The IDT introduces five influencing factors: relative advantage, compatibility, complexity, observability, and trialability. This theory is adopted in this study because it predicts the diffusion of innovations, and the application of intelligent devices in classroom behavior analysis is an innovative technology in teaching. However, since intelligent devices have not been widely available in the market, teachers primarily rely on images and videos to understand the features and usage of such devices. Therefore, trialability and observability are not included in this study. Additionally, complexity is similar to the discomfort factor in the TRI model, so complexity is replaced by discomfort.

The Technology Acceptance Model (TAM) (Davis, 1989; Alsharida et al., 2021) is the most popular and widely used model for predicting technology adoption and usage intention. It involves two key variables: perceived usefulness and perceived ease of use, which influence the intention to use technology through attitudes toward the technology (Vijayasarathy, 2004; Vorm and Combs, 2022). Perceived usefulness refers to the extent to which users believe that intelligent devices offer higher performance compared to traditional methods. Perceived ease of use refers to the extent to which users believe that intelligent devices are easy to use.

**Figure 1:**
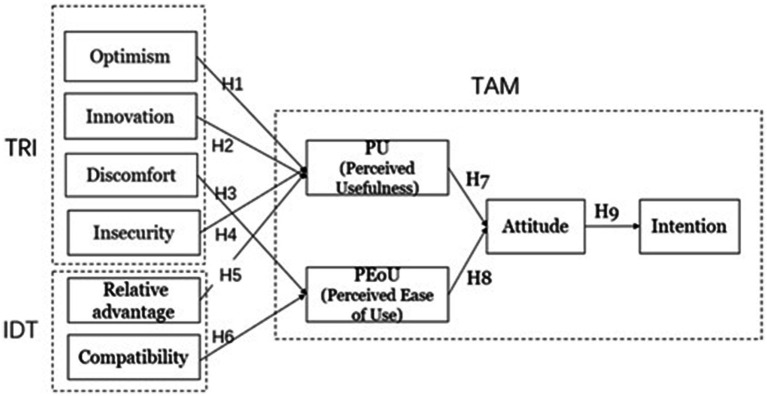
Proposed models: combination of TRI, IDT, and TAM.

However, TAM is simplistic and overlooks external factors; IDT has pro-innovation bias and assumes homogeneity; TRI focuses on individual readiness but lacks diffusion dynamics. We adopt an integrated model to avoid these limitations. In the aforementioned models, some variables are very similar, and we select one of them for study; there are also some variables that are not applicable and therefore not included in this study. The proposed model is depicted in Figure 1.

### 3.2 Hypotheses

#### 3.2.1 TRI

Technology readiness is associated with the inclination to use technology. It is determined by both the drive and inhibition factors that influence the tendency to adopt new technologies. TRI is used to measure individuals’ general beliefs about technology. The TRI theory has been widely applied in the field of education. Previous research on the use of new technologies in the education field has found significant relationships between optimism, innovativeness, discomfort, and insecurity with perceived usefulness (PU) and perceived ease of use (PEoU) (Mufidah et al., 2022; Karahoca et al., 2018; Kaushik and Agrawal, 2021).

##### 3.2.1.1 Optimism

Optimism reflects individuals’ positive views that a certain technology will empower them with more power, functionality, and productivity. Therefore, the following hypothesis is proposed:

*H1*: Optimism has a positive impact on PU when using intelligent devices in teaching.

##### 3.2.1.2 Innovativeness

Innovativeness is set as the motivation to try new technologies. Thus, the following hypothesis is proposed:

*H2*: Innovativeness has a positive impact on PU when using intelligent devices in teaching.

##### 3.2.1.3 Discomfort

Discomfort refers to the feeling of losing control over technology or being overwhelmed by it. In this case, teachers may exhibit greater resistance or skepticism toward the system, and it is expected that the intention to use intelligent devices will be weaker. Therefore, the following hypothesis is proposed:

*H3*: Discomfort has a negative impact on PEoU when using intelligent devices in teaching.

##### 3.2.1.4 Insecurity

Insecurity mainly focuses on mistrust of the technology, suspicions about its performance quality, and concerns about information security. Thus, the following hypothesis is proposed:

*H4*: Insecurity has a negative impact on PU when using intelligent devices in teaching.

#### 3.2.2 IDT

The Innovation Diffusion Theory (IDT) describes how innovations or technologies are accepted and spread in society (Rogers, 2003). It is a decision process where individuals decide whether to adopt the innovation, new service, or product. IDT also indicates that specific attributes of innovation impact consumers’ decisions to adopt it. These attributes are relative advantage, compatibility, complexity, trialability, and observability.

##### 3.2.2.1 Relative advantage

Relative advantage is the degree to which innovation is perceived as better than the idea it replaces, and it depends on individuals’ perception of the innovation being advantageous (Hashem and Tann, 2007; Al-Tkhayneh et al., 2023). It can be measured by economic, social prestige factors, convenience, and satisfaction. In this study, teachers may perceive using intelligent MR devices as more economically advantageous (e.g., lower opportunity cost for improving teaching quality), socially prestigious (e.g., aligning with important reference individuals who embrace new technologies), more convenient (e.g., easier to use than complex computer systems), and more satisfying (e.g., better experience using intelligent MR devices). When teachers have a consciousness that intelligent devices have more advantages over traditional teaching, they will have a stronger intention to use intelligent MR devices. Therefore, the following hypothesis is proposed:

*H5*: Relative advantage has a positive impact on PU when using intelligent devices in teaching.

##### 3.2.2.2 Compatibility

Compatibility refers to the extent to which individuals perceive the innovation as consistent with their lifestyle, values, past experiences, and needs (Lu and Yu‐Jen, 2009; Ayanwale and Ndlovu, 2024). Compatibility needs to be distinguished from relative advantage. Relative advantage is based on the comparison of costs and benefits. In this case, compatibility refers to the use of intelligent MR devices being in line with teachers’ existing teaching methods and styles without conflicts. Therefore, the following hypothesis is proposed:

*H6*: Compatibility has a positive impact on PEoU when using intelligent devices in teaching.

Complexity refers to the degree to which individuals perceive the innovation as “difficult to understand and use.” It is similar to the discomfort in TRI theory and replaces it in this context. Trialability and observability are not included in this study as the intelligent MR devices used for classroom behavior analysis have not been widely deployed in the market yet. Teachers mainly rely on images and videos to understand the functionalities and usage of intelligent devices.

#### 3.2.3 TAM

It is crucial to determine whether teachers will continue to use new educational technologies. In this study, attitude refers to teachers’ attitudes toward using intelligent MR devices for classroom behavior analysis. For teachers, how they perceive their ability to effectively use MR devices to improve teaching quality, especially when the device developers announce that the intelligent MR devices are designed based on teaching needs, will influence their attitude toward MR devices. Some research indicates that perceived usefulness (PU) is related to users’ attitudes toward using new technology, and attitude influences users’ behavioral intentions (Aldraiweesh and Alturki, 2025). Intelligent MR devices are not one-time use tools; they require long-term use in teaching, aiming to help teachers systematically track student classroom behavior and provide continuous support and analysis. Users need to establish an attitude of exploring intelligent technology in teaching in order to continue using new technology in their work (Bölen, 2020; Ma et al., 2025). Therefore, the following hypotheses are proposed:

*H7*: PEOU has a positive impact on users’ attitudes toward using intelligent devices.

*H8*: PU has a positive impact on users’ attitudes toward using intelligent devices.

*H9*: Users’ attitudes toward using intelligent devices in teaching have a positive impact on their intention to continue using them.

## 4 Experiment

### 4.1 Survey by questionnaire

AI serves as the core technology for data processing, personalization, and behavior analysis in educational tools, while Mixed Reality (MR) provides an interactive platform that merges virtual and physical elements. In this study, the evaluated devices are AI-integrated MR systems, where AI enables features like real-time student status tracking via facial recognition, and MR delivers augmented visualizations. The questionnaire referred to these as “intelligent MR equipment” to encompass the AI-driven functionalities.

#### 4.1.1 Development of the survey questionnaire

The research subjects are teachers from various regions and schools in China. The questionnaire is divided into three parts: The first part introduces smart devices and briefly describes their advantages, functions, and usage processes. Since respondents may not be familiar with smart devices, the first part of the questionnaire includes written explanations, five pictures, and a one-minute video file to provide information about smart devices. The second part collects socio-demographic data such as gender, teaching experience, education level, and geographical location. The third part of the questionnaire uses a seven-point semantic differential scale (Dilnozaxon, 2025), where “1” represents “completely disagree” and “7” represents “completely agree.” For more details, please refer to Supplementary Appendix A.

#### 4.1.2 Data collection and sample statistics

To test the feasibility of the questionnaire, we invited a professor, three associate professors, and five lecturers to answer the pilot questionnaire from December 11, 2023, to November 14, 2023. Feedback indicated that all respondents understood all the questions. The final questionnaire was collected from January 24, 2024, to February 2, 2024, and a total of 268 questionnaires were collected. The survey was conducted in the form of an online electronic questionnaire, and respondents were teachers from different regions contacted through social media and university alumni. The questionnaire sample included males and females from megacities and small and medium-sized cities as well as rural areas. All participants successfully completed every question in the questionnaire.

This study involves student questionnaires and has been approved by the Academic Ethics Committee of the School of Computer Science at Huazhong University of Science and Technology, adhering to the principles of the Declaration of Helsinki. The study was approved by the Academic Ethics Committee of the School of Computer Science on December 10, 2023, with the ethical review approval number (G-2023-0015-CS). After approval, we began the preparatory stage of the experiment. The designated time for the formal experiment was from January 24, 2024, to December 30, 2024.

We provided all participants with a written informed consent form. Participants were clearly informed that their participation was entirely voluntary, and they could withdraw at any time without any consequences. Additionally, this study does not involve minors.

Table 1 reports the characteristics of the respondents. The research questionnaire was collected with the following features:

1. The questionnaire was distributed online, and the interviewer was not present during the questionnaire completion process to avoid potential influence.
2. The survey was anonymous.
3. The questions in the questionnaire were concise and clear to avoid misunderstandings.

**Table 1:** Sample feature statistics.

Location	Total	Megacity	Middling/small cities	Countryside
	268	104	87	77
Gender				
Male	128	54	39	35
Female	140	50	48	42
Teaching experience				
1–2 years	69	29	22	18
3–5 years	64	23	21	20
5–10 years	73	28	24	21
Over 10 years	62	24	20	18
Subject				
Natural science	175	66	52	57
Social sciences	93	48	25	20
Educational background				
Bachelor	82	7	26	49
Master	98	41	35	22
Doctor	88	56	26	6

Based on the above points, social expectation bias can be reduced in experiments and surveys (Larson, 2019; Stantcheva, 2023).

Educational inequality is the focus of this study. When selecting respondents, we divided them into mega cities, small and medium cities, and rural areas based on their working regions, trying to select an equal number of respondents from the three types of areas. In addition, factors such as gender, teaching experience, teachers’ own educational backgrounds, and subjects taught may affect teachers’ willingness to use artificial intelligence devices, so we conducted separate statistics for these factors.

### 4.2 Control group teaching experiment

In order to further investigate the effectiveness of smart devices in practical teaching, we invited teachers and students from 12 schools to participate in teaching experiments. This study applied AI smart devices to the classroom behavior analysis of students in urban schools (4) and rural schools (8), aiming to promote the comprehensive improvement of teaching quality. The intelligent recognition system equipped with AI smart devices can capture students’ learning states in real-time and provide timely feedback to teachers, enabling them to flexibly adjust teaching strategies, thereby optimizing teaching outcomes (see Supplementary Appendix B for details).

This experiment aims to thoroughly explore the practical application effects of AI smart devices in the Chinese education sector. To this end, we selected four representative cities in China, including Shijiazhuang, Wenzhou, Taiyuan, and Xining, as well as eight rural schools located in Wenquan, Xianning, Hubei; Cishan, Wu’an, Hebei; Dazhai, Jinzhong, Shanxi; Nan’an, Quanzhou, Fujian; Shengtang, Jiangmen, Guangdong; Bikou, Longnan, Gansu; Hualong, Haiduong, Qinghai; and Shuanglin, Huzhou, Zhejiang. A three-month research sampling was conducted from the start of the fall semester (September 1, 2024) to November 30, 2024. During the sampling survey period, detailed observations and records were made regarding the teachers’ lectures and the use of AI smart devices in these schools. The study primarily focused on two courses: one in the social sciences (history) and the other in the natural sciences (mathematics). By comparing the effects of traditional teaching methods with those aided by AI smart devices, we aim to reveal the application potential and actual impact of AI technology in the field of education, in hopes of providing a scientific basis and practical guidance for future educational reform and development.

## 5 Results

### 5.1 Teacher willingness analysis

#### 5.1.1 Reliability and validity analysis

Firstly, a confirmatory factor analysis was conducted to measure the fit of the model. We also checked the effectiveness and reliability of the measurement items. Table 2 lists the model fitting indices for all structures, including the standardized factor load (*λ*), Cronbach coefficient (*α*), Mean square error (AVE), composite reliability (CR), and variance inflation factor (VIF).

1. Standardized factor loading for 11 structures (λ) Exceeding the recommended criterion of 0.5 indicates a sufficient level of reliability (Sarstedt et al., 2021).
2. Cronbach coefficient (α) Exceeding the general standard of 0.7 indicates a high reliability of the measurement items (Taber, 2018; Zakariya, 2022).
3. The average variance (AVE) of all 11 structures is higher than the standard of 0.5, indicating convergence effectiveness (Ab Hamid et al., 2017; dos Santos and Cirillo, 2023).
4. The composite reliability (CR) of all 11 structures is between 0.91 and 0.97, above the acceptable threshold of 0.7 (Liang and Chia, 2014; Lai, 2021).
5. The recommended value for VIF is less than 5 to avoid multicollinearity problems (Hair et al., 2011). The VIF values of all projects are less than 5, which meets the requirements.

**Table 2:** Analysis of reliability and effectiveness.

Variables	Item	λ	α	AVE	CR	VIF
Optimism	OP1	0.998	0.92	0.882	0.957	2.762
	OP2	0.883				1.985
	OP3	0.931				2.231
Innovation	INN1	0.894	0.86	0.842	0.955	3.245
	INN2	0.890				2.762
	INN3	0.885				2.762
	INN4	0.996				2.875
Discomfort	DISC1	0.912	0.93	0.897	0.963	2.652
	DISC2	0.985				2.380
	DISC3	0.943				2.538
Insecurity	INS1	0.899	0.89	0.816	0.93	1.042
	INS2	0.904				1.039
	INS3	0.906				2.525
Relative advantage	RLA1	0.991	0.94	0.882	0.968	3.770
	RLA2	0.870				2.589
	RLA3	0.929				2.840
	RLA4	0.963				2.224
Compatibility	CP1	0.800	0.92	0.833	0.937	3.061
	CP2	0.958				4.114
	CP3	0.970				2.209
PU	PU1	0.862	0.86	0.767	0.908	1.879
	PU2	0.864				4.699
	PU3	0.901				2.816
PEoU	PEoU1	0.950	0.91	0.854	0.946	2.658
	PEoU2	0.864				2.869
	PEoU3	0.956				2.855
Attitude	AT1	0.884	0.89	0.808	0.926	3.273
	AT2	0.948				4.454
	AT3	0.861				3.647
Intention	IN1	0.894	0.87	0.827	0.934	3.133
	IN2	0.831				2.094
	IN3	0.994				2.727

The results indicate that the measurement model has sufficient fit and the measurement items are reliable and effective.

#### 5.1.2 Hypothesis testing

We established a structural model of partial least squares regression to examine the relevance of all 11 hypotheses. The results are shown in Table 3.

**Table 3:** Hypothesis testing.

Hypothetical relationship	β	*SE*	*t*-test	*p*	Results
H1: Optimism - > PU	0.270	0.074	3.430	0.001	Supported
H2: Innovation - > PU	0.231	0.078	3.347	0.010	Supported
H3: Discomfort - > PEoU	−0.671	0.082	−10.299	0.000	Supported
H4: Insecurity - > PU	−0.215	0.083	−2.603	0.009	Supported
H5: Relative advantage - > PU	0.400	0.081	4.137	0.000	Supported
H6: Compatibility - > PEoU	0.365	0.089	5.766	0.000	Supported
H7: PU - > Attitude	0.595	0.055	4.565	0.000	Supported
H8: PEoU - > Attitude	0.124	0.039	1.896	0.084	Not supported
H9: Attitude - > Intention	0.950	0.078	8.839	0.000	Supported

*β*, Standardized regression coefficient; SE, Standard error.

The results indicate that optimism, innovation, and comparative advantage exhibit a positive relationship with PU. Therefore, supports H1, H2, and H5.

Discomfort (*β* = − 0.671, *t* = −10.299, *p* < 0.05) has a significant relationship with PEoU and has a negative impact, supporting H3.

Insecurity (*β* = − 0.215, *t* = −2.063, *p* < 0.05) has a significant relationship with PU and has a negative impact, supporting H4. Compatibility (*β* = 0.365, *t* = 5.766, *p* < 0.05) is positively correlated with PU, supporting H6.

In addition, expected confirmation (*β* = 0.814, *t* = 9.380, *p* < 0.05) and PU (*β* = 0.595, *t* = 4.565, *p* < 0.05) has a significant relationship with attitude, supporting H7.

There is no significant relationship between perceived ease of use and attitude (*p* > 0.05), and H8 is not supported. Attitude and intention (*β* = 0.950, *t* = 8.839, *p* < 0.05) is positively correlated, supporting H9.

#### 5.1.3 Validity of measurements

Content validity was addressed during the initial design phase of the questionnaire. The items for each construct were adapted from seminal works and established scales within the TRI, IDT, and TAM literature, ensuring a strong theoretical foundation. Furthermore, to ensure the clarity, relevance, and comprehensibility of the items within the specific context of AI-integrated MR devices in Chinese education, the questionnaire underwent a pilot test. We solicited feedback from a panel of nine academic experts (one professor, three associate professors, and five lecturers), who confirmed that the questions were well-understood and appropriate for the target teacher population.

The validity of our measurement instruments extends beyond the internal structure evidenced by CFA (Table 2), incorporating content validity through expert review and pilot testing, which ensured items accurately represented TRI, IDT, and TAM constructs in the rural AI education context. This approach parallels content validation in related studies, such as Rui et al. (2023), who adapted TAM for Chinese university professors’ AI adoption and reported high expert agreement (CVI = 0.89). Criterion-related validity is suggested by the model’s predictive links to teachers’ intentions (e.g., H9: *β* = 0.950), correlating with real-world outcomes like sustained device use, consistent with Alhasan et al. findings on IoT in smart classrooms (*r* = 0.58 for PU-Intention) (Alhasan et al., 2023). However, we did not conduct full predictive or concurrent validity tests against external criteria (e.g., longitudinal adoption rates), which limits generalizability compared to multi-wave designs (Teng et al., 2022). Future research could incorporate such assessments to further validate the composite model across diverse educational settings.

### 5.2 Control group teaching experiment

To systematically evaluate the actual effectiveness of AI smart devices in classroom teaching, classes with comparable basic levels were selected for controlled experiments in schools across the chosen regions. The experimental group classes introduced intelligent teaching devices for full-subject instruction, while the standard group classes followed conventional teaching models without any involvement of smart devices. After rigorous sample selection and data collection, detailed teaching data from 8 experimental group classes and 8 standard group classes were successfully obtained. Next, this study will conduct a meticulous statistical analysis of this data, aiming to precisely reveal the differences and potential impacts between the two types of classes in terms of using AI smart devices.

Class scores for learner effectiveness were collected via standardized end-of-term examinations to ensure comparability across urban and rural schools. China’s 9-year compulsory education system mandates unified textbooks and consistent learning content, providing a standardized curriculum framework. To facilitate observation of teaching effectiveness, all participating schools, with consent from local education departments, administered uniform final exam test papers for mathematics (natural sciences) and history (social sciences) courses. These test papers, developed in collaboration with educational experts, included multiple-choice, short-answer, and problem-solving sections aligned with the national curriculum covered during the three-month period (September 1, 2024, to November 30, 2024). Pre-experiment baseline assessments confirmed that experimental and standard classes had comparable starting proficiency levels (average baseline scores differed by less than 5% within each school). Post-experiment scores were anonymized, graded by independent evaluators blind to group assignments, and aggregated to calculate average class performance. This approach minimized biases from regional variations in teaching styles or resources, ensuring direct comparability of AI-assisted versus traditional teaching methods across all settings.

The following experimental data charts visually display the differences in teaching effects after using AI smart devices between experimental and standard classes in urban and rural schools.

Tables 4, 5 show the experimental results of the control group teaching in four urban schools. The average score for Natural Sciences (Mathematics) in the experimental class is 92.11, and for Social Sciences (History) it is 88.55; in the standard class, the average score for Natural Sciences (Mathematics) is 83.01, and for Social Sciences (History) it is 80.83. The overall average score of the experimental class is 10.27% higher than that of the standard class.

**Table 4:** Comparison of the teaching effectiveness of mathematics courses in four urban schools.

Mathematics	A Shijiazhuang school	B Wenzhou school	C Taiyuan school	D Xining school	Average score
Experimental class	93.56	96.23	90.48	88.15	92.11
Standard class	85.79	87.90	80.23	78.11	83.01
Improved score	7.77	8.33	10.25	10.04	9.10
Proportion of increase	9.06%	9.48%	12.78%	12.85%	10.96%

**Table 5:** Comparison of the teaching effectiveness of history courses in four urban schools.

History	A Shijiazhuang school	B Wenzhou school	C Taiyuan school	D Xining school	Average score
Experimental class	91.89	90.23	85.65	86.43	88.55
Standard class	85.51	83.45	76.20	78.16	80.83
Improved score	6.38	6.78	9.45	8.27	7.72
Proportion of increase	7.46%	8.12%	12.40%	10.58%	9.55%

Tables 6, 7 show the experimental results of the control group teaching in eight rural schools. The average score for Natural Sciences (Mathematics) in the experimental class is 93.00, and for Social Sciences (History) it is 88.35; in the standard class, the average score for Natural Sciences (Mathematics) is 78.86, and for Social Sciences (History) it is 77.87. The overall average score of the experimental class is 15.69% higher than that of the standard class.

**Table 6:** Comparison of the teaching effectiveness of mathematics courses in eight rural schools.

Mathematics	E	F	G	H	I	J	K	L	Average score
Experimental class	92.12	93.46	95.31	94.38	96.12	93.50	87.34	91.76	93.00
Standard class	78.25	75.67	76.37	77.52	86.39	81.80	76.45	78.45	78.86
Improved score	13.87	17.79	18.94	16.86	9.73	11.70	10.89	13.31	14.14
Proportion of increase (%)	17.73	23.51	24.80	21.75	11.26	14.30	14.24	16.97	17.93

E, Hubei Xianning Wenquan; F, Hebei Wu’an Cishan; G, Guangdong Jiangmen Shengtang; H, Fujian Quanzhou Nan’an; I, Shanxi Jinzhong Dazhai; J, Gansu Longnan Bikou; K, Qinghai Haiduong Hualong; L, Zhejiang Huzhou Shuanglin.

**Table 7:** Comparison of the teaching effectiveness of history courses in eight rural schools.

History	E	F	G	H	I	J	K	L	Average score
Experimental class	89.25	90.89	91.12	88.67	87.63	85.10	81.56	92.57	88.35
Standard class	80.34	78.98	81.86	77.34	82.74	71.59	69.78	80.33	77.87
Improved score	8.91	11.91	9.26	11.33	4.89	13.51	11.78	12.24	10.48
Proportion of increase (%)	11.09	15.08	11.31	14.65	5.91	18.87	16.88	15.24	13.46

The meaning of E-L is the same as in Table 6.

The results indicate that the teaching effectiveness of the experimental class, which used AI smart devices, was significantly better than that of the standard class. This finding confirms the effectiveness of smart devices in the field of education, specifically reflected in the fact that the average scores of the experimental class were noticeably higher than those of the standard class that did not use smart devices. This data not only highlights the immense potential of AI technology in improving teaching quality but also provides strong empirical support for future educational reforms and innovations.

**Figure 2:**
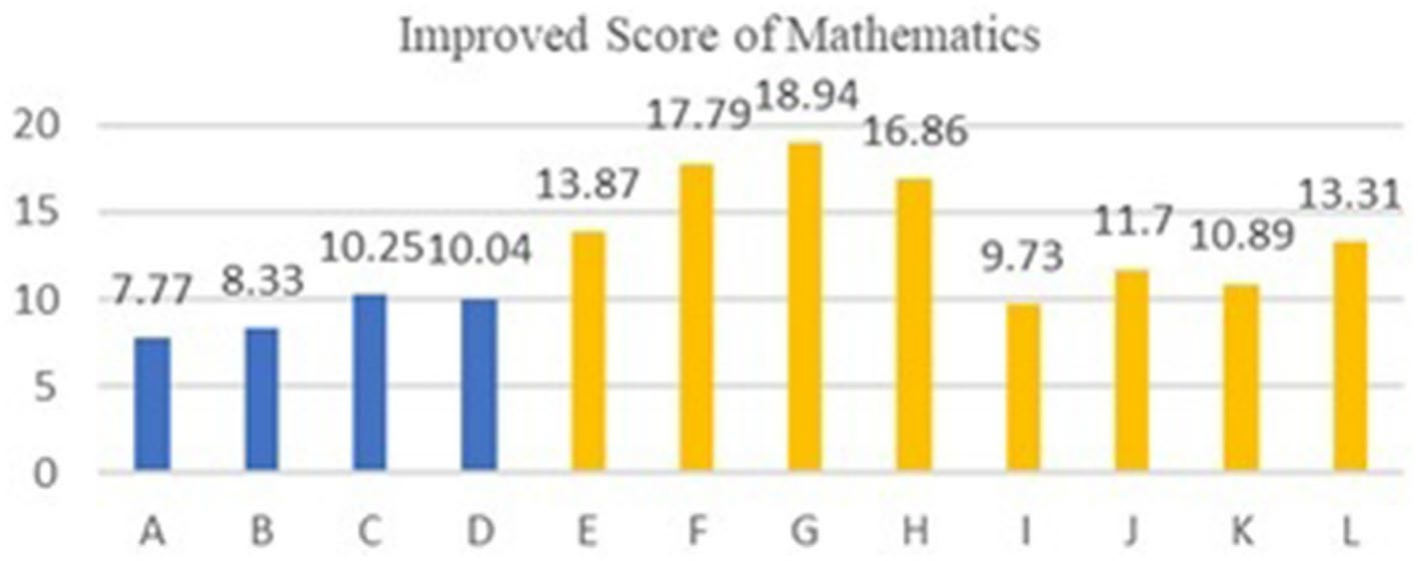
Comparison of the improvement in mathematics course between experimental classes and standard classes in 16 schools. Urban school, A: Shijiazhuang school, B: Wenzhou School, C: Taiyuan School, D: Xining School. Rural school, E-L is the same as in Table 6.

**Figure 3:**
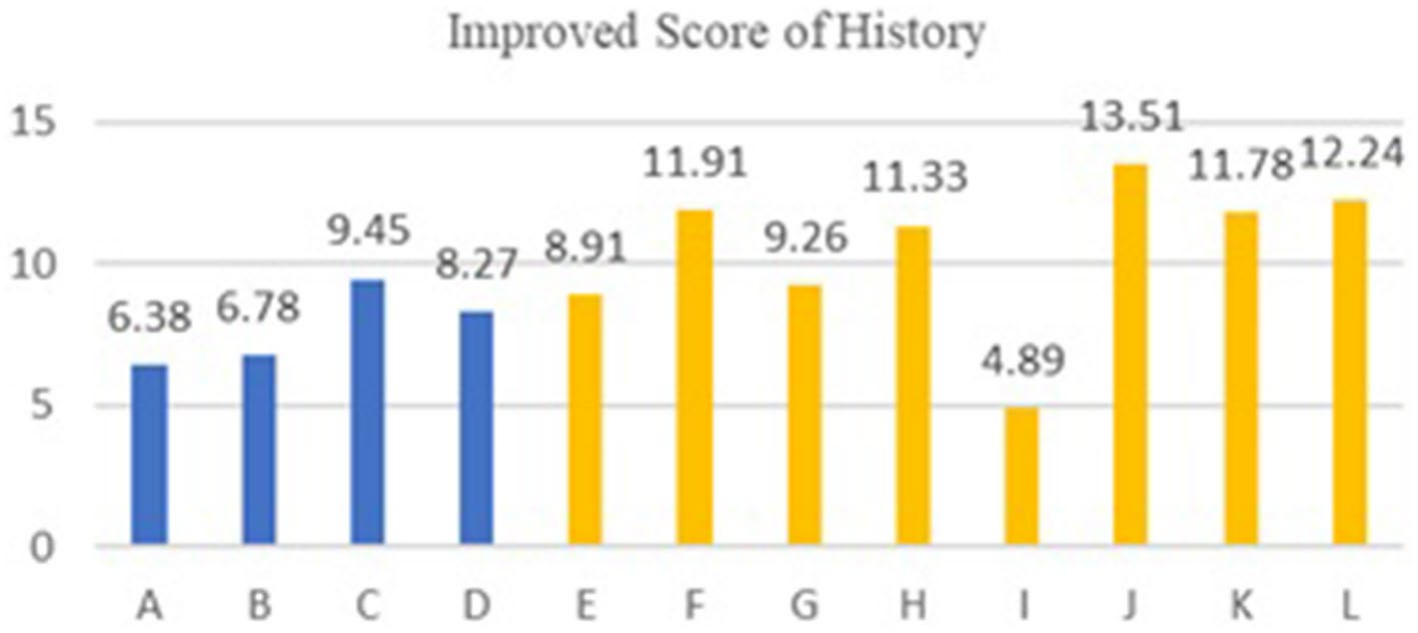
Comparison of the improvement in history course between experimental classes and standard classes in 16 schools. The meaning of A-L is the same as in Figure 2.

We compared the improved scores of experimental classes in rural schools with those in urban schools, and the improved scores of mathematics and history classes are shown in Figures 2, 3, respectively. The average score for improving mathematics courses in urban schools is 9.10, while the average score for improving mathematics courses in rural schools is 14.14. The effectiveness of rural schools is 55.4% higher than that of urban schools. The average score for improving history courses in urban schools is 7.72, while the average score for improving history courses in rural schools is 10.48. The effectiveness of rural schools is 35.7% higher than that of urban schools. The results show that AI has a noticeable effect in Natural Sciences courses due to their high demand for coherence, which can improve learning efficiency, while its effect is weaker in Social Sciences courses, as their demand for coherence is lower.

## 6 Discussion

### 6.1 Analysis of experimental results

The research results indicate that teaching methods assisted by AI smart devices are significantly superior to traditional teaching methods, which not only reflects the advancement of educational technology but also aligns with the concept of sustainable development. In both natural science and social science courses, the average scores of the experimental classes using AI devices were higher than those of the standard classes taught traditionally, demonstrating the potential of AI technology in enhancing the quality and efficiency of education. Especially in rural schools where resources are relatively scarce, the introduction of AI devices greatly improved teaching effectiveness. This not only helps to narrow the urban–rural educational gap and promote educational equity but also has significant importance for achieving sustainable development in education, highlighting the unique value of AI technology in these environments and its positive impact on future educational models.

In promoting educational equity, the data provides strong support. The experiment shows that the personalized and data-driven teaching support of AI technology helps to narrow the urban–rural educational gap. By using AI smart devices, students in rural schools can access high-quality educational resources comparable to those in urban areas, significantly enhancing learning outcomes and teaching quality.

Furthermore, the research also highlights the transformation of the teacher’s role and professional development. In the AI-assisted teaching environment, the role of teachers gradually shifts from traditional knowledge transmitters to facilitators and promoters of learning. The real-time feedback and data analysis provided by AI devices help teachers gain a deeper understanding of students’ learning needs, optimize teaching strategies, and promote their professional growth.

### 6.2 Contribution of research

#### 6.2.1 Educational equity

Educational equity aims to ensure that every student, regardless of their socio-economic background, race, gender, or geographic location, has equal access to educational opportunities and resources. This concept not only focuses on the equal distribution of resources but also emphasizes recognizing and respecting students’ individual differences to help each student achieve their maximum potential.

From the perspective of educational equity, the effectiveness demonstrated by rural schools after adopting AI smart devices in teaching is particularly striking. The theory of educational equity emphasizes that all students should have equal educational opportunities and resources (Lucas and Beresford, 2010), unrestricted by geographic location. Although rural schools have relatively fewer teaching resources and equipment, this has not prevented them from showing enthusiasm and effectiveness in using AI smart devices. This indicates that in resource-constrained environments, the application of technology can significantly improve the quality and accessibility of education.

AI smart devices, through their ability to share resources and expand functionality, enable rural students to access high-quality educational resources on par with those available to urban students, helping to bridge the urban–rural educational gap. For example, through interactive learning platforms and online resources, rural students can access advanced educational content and teaching methods that are typically provided only in resource-rich urban schools.

Furthermore, in the process of adopting AI smart devices, rural schools usually place greater emphasis on meeting the personalized needs of students. The flexibility of AI technology allows teachers to provide customized teaching plans based on the specific needs of students, thereby enhancing the effectiveness and appeal of teaching. At the same time, teachers can not only improve teaching efficiency through interaction with AI devices but also enhance their teaching skills and professional knowledge with the feedback and data support provided by the devices.

Over the past decade, global experiments and initiatives in educational equity have made significant progress by integrating modern technology and innovative educational concepts. For instance, the “One Laptop per Child” (One Laptop per Child, 2009; Li et al., 2022) project continues to promote personal computers in developing countries to improve students’ computer skills and academic performance, despite challenges in maintenance and training. “Success Academy Charter Schools” in New York have demonstrated that significant academic achievements can be realized even in resource-limited environments by providing rigorous curricula and high-standard teaching, supporting the concept of educational equity (Cohodes and Parham, 2021). In Africa, “Bridge International Academies” offer affordable education to students from low-income families through standardized curricula and technology-driven teaching management, with students performing well in standardized tests (Riep, 2017; Walker et al., 2022). The “Schoolhouse world” project by Khan Academy provides free live group tutoring, allowing students worldwide access to high-quality educational resources (Khan, 2012; Bhaw et al., 2024). These cases have proven that the integration of innovative methods and technology can effectively promote educational equity and enhance the quality and accessibility of global education.

Therefore, the significant improvement in teaching effectiveness in rural schools after adopting AI smart devices reflects the combined effects of the balanced allocation of educational resources, the emphasis on personalized teaching, and the enhancement of teachers’ professional qualities. These changes not only support the realization of educational equity but also demonstrate the potential of educational technology in promoting fairness. In the future, further attention should be paid to the needs and challenges of rural schools in the development of educational informatization, continue to promote the in-depth implementation of educational equity strategies, and ensure that every student can benefit from the educational opportunities brought about by technological advancements.

#### 6.2.2 Educational efficiency

The theory of educational efficiency focuses on how to achieve the maximum educational output with the least input of resources, including analyzing the cost-effectiveness of teacher salary increases and investments in educational facilities, as well as how these investments can effectively improve educational outcomes.

In the field of education, the theory of teaching efficiency emphasizes maximizing resource utilization and optimizing teaching outcomes during the teaching process (Baer, 1939; Permatasari and Suryadi Tandiayuk, 2023). According to this theory, teaching in the field of natural sciences, after the adoption of AI smart devices, usually has better teaching efficiency and effectiveness than social sciences teaching. This phenomenon can be explained from multiple perspectives. First, teaching in natural sciences often involves a large number of experiments, observations, and data analysis, which are activities well-suited for assistance with AI smart devices. AI devices can provide real-time data analysis and experimental result presentations, helping students to understand complex concepts more intuitively, thereby improving teaching efficiency. At the same time, AI devices can also automatically grade experimental reports and data, saving teachers’ time and allowing them to focus more on personalized guidance for students.

Secondly, the application of AI smart devices in natural sciences teaching can provide a more vivid and interactive learning experience. For example, through virtual reality (VR) and augmented reality (AR) technologies, students can conduct scientific explorations and experimental operations in virtual environments. This not only increases students’ interest in learning but also enhances the intuitiveness and practicality of teaching outcomes.

In recent years, the application of AI technology in the field of natural sciences teaching has achieved significant results, especially in improving teaching efficiency and optimizing learning experiences. For instance, the online learning platform EdX (Shi and Lin, 2021), developed jointly by MIT and Harvard University, has significantly improved students’ learning outcomes in subjects such as physics and chemistry by providing personalized learning paths and automated feedback systems. Students can adjust the difficulty of the course according to their own learning pace, thereby more effectively mastering complex scientific concepts. Another example is the AI-assisted chemistry laboratory system developed by Stanford University, which can analyze students’ experimental data in real-time and provide immediate feedback, helping students to deeply understand chemical reactions and improve their experimental skills (Zaman et al., 2021). Students using this system have significantly improved their grades in chemistry courses, and teachers can also use the data provided by AI to optimize teaching strategies and content. These innovative teaching tools not only improve the quality of teaching but also bring new development directions to the field of education, demonstrating the immense potential and practical application value of AI technology in education.

From the perspective of the theory of teaching efficiency, the introduction of AI smart devices helps to achieve a balanced distribution of teaching resources. In traditional teaching, natural sciences teaching often faces limitations due to experimental equipment and materials, making it difficult to implement comprehensive experimental teaching. The widespread adoption of AI smart devices allows these resources to break through geographical and temporal constraints, providing more students with high-quality experimental teaching opportunities, thereby narrowing the gap between urban and rural areas and levels of education.

In the future, there should be continued exploration of the potential applications of AI smart devices in teaching different subjects, promoting a balanced distribution of educational resources and further optimization of teaching outcomes.

### 6.3 Limitations and future research

This study has confirmed the effectiveness of AI smart devices in improving educational quality and promoting educational equity, especially their great potential in rural schools with fewer resources, which aligns with the core concept of sustainable development—promoting social justice and the rational allocation of resources. Future research should further explore the effects of AI technology in different teaching subjects and specific application scenarios, particularly the differences in application between courses such as natural sciences and social sciences, and how to achieve sustainable education through technological means. At the same time, research should focus on how to effectively train teachers to adapt to technology-driven educational environments, especially conducting detailed analysis in terms of teaching styles and technical proficiency to promote professional growth of teachers and the overall improvement of educational quality.

There are still some limitations to this study. First, the sample size is relatively small, which may limit the generalizability of the findings. Future studies should consider larger and more diverse samples to validate our results. Furthermore, the study focuses on the educational impact of AI devices and does not provide a comprehensive economic analysis. Future research should include economic evaluations to assess the cost-effectiveness and long-term benefits of AI devices in rural educational settings. Thirdly, while this study established validity through content review and an analysis of the instrument’s internal structure and theoretical relationships, it did not assess criterion-related validity. The survey measured teachers’ self-reported intentions and attitudes, but these were not correlated with an external, objective criterion, such as data logs of their actual device usage or observed teaching behaviors. Future studies could strengthen the validity evidence by comparing survey responses with objective behavioral data to see if the expressed intentions translate into practice.

Targeting deeper segmentation of student characteristics, such as learning motivation, family background, and their correlation with AI teaching effectiveness, is also an important direction for future research. This includes how to design personalized AI educational content to meet the needs of different students, ensuring that every student can benefit from it, thereby promoting the development of educational equity and social inclusiveness.

Finally, focusing on the long-term sustainability and potential social impacts of AI technology in education, including the long-term effects on educational equity and adaptability in different socio-economic environments, will provide a scientific basis and practical guidance for future educational reforms and development, promoting the balanced and sustainable development of the educational cause.

## 7 Conclusion

The present study has delved into the necessity and impact of integrating AI devices into rural schools within the framework of sustainable development. Through a comprehensive analysis, the research has underscored the transformative potential of AI in addressing the multifaceted challenges faced by rural education, particularly in regions with limited resources. The study’s findings reaffirm that AI devices, when strategically implemented, can significantly enhance teaching quality, improve learning outcomes, and contribute to educational equity.

The application of AI in rural educational settings has been found to be particularly impactful in natural science courses, where the integration of AI devices has led to marked improvements in teaching efficiency and student engagement. The study’s experimental results, supported by questionnaire surveys and regression analysis, provide robust evidence that AI-assisted teaching can bridge the urban–rural divide in education, offering a more level playing field for students regardless of their geographical location.

Moreover, the study emphasizes the importance of considering various factors that affect teachers’ acceptance of smart devices, including technological readiness, innovation diffusion, and technology acceptance. The proposed comprehensive model, which integrates TRI, IDT, and TAM, offers a nuanced understanding of the factors influencing the adoption of AI devices in education.

Looking ahead, the study calls for further research to explore the application effects of AI technology across different subjects and to strengthen teacher training and technical support. This will ensure that AI devices can be effectively utilized to achieve educational equity and sustainable development goals. The research also suggests that as AI technology continues to evolve, its role in enhancing educational outcomes and narrowing the gap between urban and rural schools will become increasingly significant.

## Data Availability

The raw data supporting the conclusions of this article will be made available by the authors, without undue reservation.
