# Supplementary material for: Advancing educational equity in rural China: the impact of AI devices on teaching quality and learning outcomes for sustainable development
Source: Front Psychol. 2025 Nov 5;16:1588047. doi: 10.3389/fpsyg.2025.1588047 (PMC12626958; doi:10.3389/fpsyg.2025.1588047)
Supplement: Supplementary file 1 [file Table_1.docx]

Supplementary Material

# Appendix A

| **Items** | **Source** |
| --- | --- |
| **Optimistic** | [44, 47] |
| I hope I can use intelligent MR devices in the classroom. |  |
| I am excited about the intelligent MR equipment in the classroom. |  |
| I think using smart portable devices in the classroom can bring me many benefits. |  |
| **Innovation** | [44, 47] |
| My friend wants to learn about smart portable devices from me. |  |
| Generally, I am the first person in my friend group to acquire new technologies when they arrive. |  |
| I have always been interested in learning new technologies. |  |
| In my field of interest, I am always able to keep up with the latest technologies. |  |
| **Insecurity** | [44, 46] |
| I think using intelligent MR devices in the classroom is not safe. |  |
| I'm worried about the information you sent through your intelligent MR device. |  |
| I have no confidence in using intelligent MR devices in the classroom. |  |
| **Discomfort** | [44, 47] |
| Occasionally, I believe that using intelligent MR devices in classrooms is not designed for the general public. |  |
| Intelligent MR device manuals may not be easy to understand. |  |
| I am confused about the amount of information I need to know when using intelligent MR devices. |  |
| **Relative advantages** | [39, 40] |
| I think using intelligent MR devices can improve my teaching effectiveness. |  |
| I think using intelligent MR devices makes it easier to improve teaching quality. |  |
| I think using intelligent MR devices enables me to improve teaching quality faster. |  |
| I think using intelligent MR devices is beneficial. |  |
| **Compatibility** | [39, 40] |
| The use of intelligent MR devices in the classroom will be compatible with my daily teaching. |  |
| Intelligent MR device technology will be suitable for my teaching method. |  |
| The use of intelligent MR device in the classroom will suit my teaching style. |  |
| **PEoU** | [35, 39, 72] |
| Using intelligent MR devices in the classroom requires no effort. |  |
| I will be able to use intelligent MR devices in the classroom. |  |
| The intelligent MR equipment in the classroom is easy to use. |  |
| **PU** | [35, 39, 72] |
| The use of intelligent MR equipment in the classroom can help improve efficiency. |  |
| The use of intelligent MR equipment in the classroom helps improve my teaching effectiveness. |  |
| Overall, I think using intelligent MR portable devices in the classroom is very useful. |  |
| **Attitude** | [45] |
| I find it interesting to use intelligent MR devices. |  |
| I look forward to using intelligent MR devices in teaching. |  |
| Overall, my attitude towards using intelligent MR devices in the classroom is that they are very useful. |  |
| **Intention** | [45] |
| I hope to use intelligent MR devices in classrooms in the near future. |  |
| I am determined to continue using intelligent MR devices in the classroom. |  |
| I plan to use intelligent MR devices in the classroom as soon as possible. |  |

# Appendix B

Recommendations for Integrating Smart Mixed Reality (MR) Devices into the Classroom

Classroom behavior analysis is challenging in traditional teaching. Teachers primarily deliver classroom lessons in a one-to-many format, making it difficult for teachers to evaluate each student's classroom performance in-depth and comprehensively. In classroom teaching, teachers rely on observing students' facial expressions or behavioral states to obtain teaching feedback. However, students' behavior in the classroom is complex and constantly changing. Teachers' primary focus is on teaching, making it challenging to fully understand students' classroom situations. This hinders the improvement of teaching quality and students' development[73].

Li[74] proposed a wearable Mixed Reality (MR) device for classroom behavior analysis, enabling teachers to receive real-time feedback on students' learning statuses and make timely adjustments to enhance teaching effectiveness.


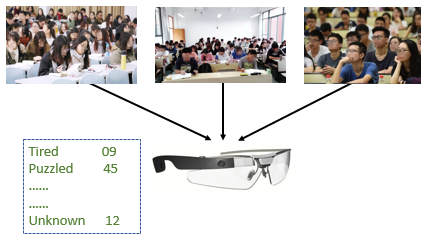


**Figure A.** Scenarios of MR equipment used for classroom behavior analysis.

The usage scenario of the MR device is illustrated in Figure A. The camera of the MR device captures real-time images, which are then transmitted to the embedded system. The AI model in the embedded system of the MR device utilizes facial emotion recognition to determine the learning status of each student and displays real-time statistics of students' learning statuses in the current classroom. This enables teachers to understand the learning statuses of students and make timely adjustments to enhance teaching quality. For example, when students show signs of confusion, it indicates a lack of understanding of the teaching content, and teachers can ask appropriate questions and provide repeated explanations. When students exhibit signs of fatigue, teachers can introduce lighter topics to relax and ease the atmosphere. When students display signs of focused seriousness, it indicates good attention and effective teaching. The intelligent MR glasses with a classroom behavior analysis model help teachers understand students' emotions and make real-time adjustments accordingly. In a control group teaching experiment, the class using the intelligent MR device achieved better teaching results.

The recommendations for integrating smart mixed reality (MR) devices into the classroom aim to provide educational institutions, educators, and developers with a clear roadmap for successful implementation. Their purpose is twofold: to promote the adoption of MR technology by providing structured guidance and support, and to enhance educational outcomes by making learning more engaging, interactive, and accessible. By addressing challenges such as teacher training, technological accessibility, and curriculum integration, these recommendations aim to empower educators to tailor educational experiences according to different contexts and foster collaboration among stakeholders. This holistic approach ensures the effective use of MR technology to enrich the educational landscape.

It is important to note that these recommendations highlight the potential of MR devices to transform education, advocating for customized solutions that consider local teaching practices and needs, especially in resource-constrained areas. They emphasize the importance of promoting educational equity through technology and encourage interdisciplinary collaboration to continuously innovate and improve MR applications. Doing so not only helps to improve the quality of education but also paves the way for the future, where technology and education work together to prepare students for the complexities of the modern world, ensuring that the transformative power of MR technology is realized across the entire educational sector.

1. Strengthen Teacher Training and Support

To facilitate the seamless integration of smart MR devices into the classroom, comprehensive teacher training programs must be established. These programs should not only focus on the technical aspects of using MR devices but also integrate teaching strategies that leverage the unique features of MR technology to enhance learning outcomes. Practical workshops that simulate real classroom scenarios, coupled with ongoing professional development opportunities, can greatly increase teachers' confidence and proficiency with these technologies. Additionally, creating a collaborative platform where educators can share their experiences, challenges, and best practices can foster a community of practice that promotes collective learning and innovation in the use of MR in education.

2. Customization According to Local Conditions

Significantly enhancing the effectiveness of MR technology in education can be achieved by customizing its applications according to the specific conditions of different regions, especially remote areas. Developers should collaborate with local educators to understand their unique teaching styles, challenges, and the specific needs of their students. This contextual understanding can inform the design of MR content and applications, making them more relevant to students and more accessible to students in different settings. By customizing MR experiences according to local cultures, languages, and educational standards, developers can ensure that these technologies have a broader and more meaningful impact, potentially transforming education in resource-constrained areas.

3. Promote Educational Equity

Smart MR devices have tremendous potential in democratizing access to high-quality education. By leveraging these technologies, educational institutions can provide immersive and interactive learning experiences that were previously inaccessible to students in resource-constrained areas. This approach not only increases student engagement and motivation but also helps create a fair educational environment. Strategic partnerships between educational authorities, non-governmental organizations, and technology providers can facilitate the deployment of MR devices in schools that are most likely to benefit, thereby addressing educational disparities and promoting equity across the educational sector.

4. Interdisciplinary Collaboration

The development and integration of smart MR devices in education should be a collective effort of educators, technicians, researchers, and even students. This interdisciplinary approach ensures that MR technologies are not only technologically advanced but also pedagogically effective and aligned with current and future educational needs. Collaboration can take many forms, from joint research and development projects to educator-technician partnerships in classroom environments. Through collaboration, stakeholders can ensure that MR devices are continuously improved and adjusted to serve as powerful tools for fostering innovative learning environments.

Implementing these expanded recommendations requires the joint efforts of all stakeholders in the educational ecosystem. By focusing on teacher training, customization according to local conditions, promoting educational equity, and fostering interdisciplinary collaboration, the integration of smart MR devices with education can be optimized to improve teaching quality and enrich students' learning experiences, ultimately contributing to the broader goals of modern education.
